# mGem: Guides or triggers? Extracellular RNAs beyond vesicular miRNAs

**DOI:** 10.1128/mbio.03129-24

**Published:** 2025-09-16

**Authors:** Juan Pablo Tosar, Amy H. Buck

**Affiliations:** 1 Functional Genomics Laboratory, Institut Pasteur de Montevideo 123939, Montevideo, Uruguay; 2 Analytical Biochemistry Unit, Center for Nuclear Research, School of Science, Universidad de la República 56724, Montevideo, Uruguay; 3 Institute of Immunology & Infection Research, School of Biological Sciences, University of Edinburgh, Edinburgh, United Kingdom; Instituto Carlos Chagas, Curitiba, Brazil

**Keywords:** exRNA, exosomes, intercellular communication, extracellular tRNAs, extracellular miRNAs, extracellular vesicles, nonvesicular RNA, TLR signaling

## Abstract

Despite a huge expansion in the last decades, several assumptions have directed, and perhaps pigeonholed, the evolution of the extracellular RNA (exRNA) field. For example, extracellular vesicles (EVs) have been assumed to be the main carriers of RNA molecules between cells. In parallel, microRNAs (miRNAs) have been assumed to be the main EV RNA cargo. However, from mammals to microbes, these assumptions do not seem to fall out of the data. In addition, miRNAs need to localize to the cytosol to be active but are likely to start in endosomes in most EV entry pathways. The mechanisms for their endosomal escape and the quantities of imported miRNAs required for their functions are not always considered. Without questioning the empirical evidence supporting EV-miRNA-mediated intercellular communication, we would like to shed light on the overlooked aspects of the exRNA biology that may bear important insights into how cells and organisms interact and sense one another.

## PERSPECTIVE

Blood and other extracellular biofluids have a high RNase content. Hence, from its origins, the exRNA field has focused on extracellular vesicles (EVs) as the main carriers of RNA molecules that move between cells (1–3). In parallel, an underlying assumption has been that microRNAs are the most important class of RNA in EVs.

However, most exRNAs are not associated with EVs (4–10). In addition, even the most abundant miRNAs are present at less than 0.1–0.01 copies per EV (7, 11–14). Other noncoding RNAs, such as tRNAs, Y RNAs, rRNAs, and their fragments, are consistently reported to be much more abundant (7, 8, 11, 14, 15).

The same qualitative conclusions can be drawn in other eukaryotes. For example, while parasitic nematodes release miRNAs in EVs (16), across multiple nematode species, miRNAs are under-represented in the extracellular space compared to other RNA biotypes (17–19). Plants also release EVs containing miRNAs and siRNAs that can silence specific genes in fungal pathogens (20). However, at least in *Arabidopsis*, both the apoplastic fluid and the leaf surface contain a heterogeneous population of exRNAs that are predominantly nonvesicular (21–23). Mammalian fungal pathogens like *H. capsulatum* release EVs that contain miRNA-like sequences but, mostly, tRNA (24). *Trypanosoma cruzi*, a parasitic protozoan that lacks miRNAs, releases EVs that mostly contain rRNA- and tRNA-derived fragments (25, 26). Similarly, in bacteria (which also do not encode miRNAs), different exRNAs have been found both inside and outside of EVs (27), including fragments of tRNAs proposed to shuttle to other cells where they act as guides to regulate gene expression (28). Recent work characterizing exRNAs in Archaea also suggests that the most abundant class is, again, tRNAs (29).

Awareness that vesicles are not the only complexes that can protect and shuttle RNAs in or out of cells has increased in recent years. For example, nonvesicular RNAs, including Argonaute 2/miRNA complexes (4, 9), have recently gained attention, thanks to the report of membraneless extracellular nanoparticles, such as exomeres and supermeres (10, 30). Nonvesicular Argonautes complexed with siRNAs are also released from parasitic nematodes and can enter host cells (31). Other large ribonucleoprotein complexes, such as extracellular ribosomes (8), can also give place to stable protein/RNA complexes upon extracellular fragmentation (32). In addition, some RNAs might achieve extracellular stability and abundance, thanks to their compact three-dimensional structures stabilized mostly by RNA:RNA interactions (33, 34). These stable “naked” RNAs can also be internalized and sensed by immune cells, playing a role in intercellular communication, especially in the context of inflammation (35). In retrospect, the assumption that all extracellular samples are characterized by a potent RNase activity might be a generalization of experiments done in either blood or in cell culture, which usually contains serum (e.g., FBS) as an additive. When naked extracellular RNA is added to physiological compartments with a low RNase content, such as the peritoneal cavity, its capacity to trigger inflammatory responses becomes evident (35).

Thus, the popularity of EVs and miRNAs is better explained by the chronology of the exRNA field and the large focus on mammalian systems, rather than emerging from the data itself. For example, the discovery of EV-miRNAs occurred at a time when the interest in miRNAs as gene expression regulators was at its peak, bolstered by a mechanistic framework for how miRNAs can regulate gene expression. A few years afterward, next-generation sequencing became widely accessible, and, consequently, many studies started to explore the small RNA content of EVs. However, small RNA sequencing was designed for miRNA identification and is highly biased toward this small RNA class (36). Without enzymatic treatment of RNAs, or modifications to protocols to read through structured RNAs, many of the exRNAs present in a sample are never detected.

What is, then, the picture that emerges from the data?

**Fig 1:**
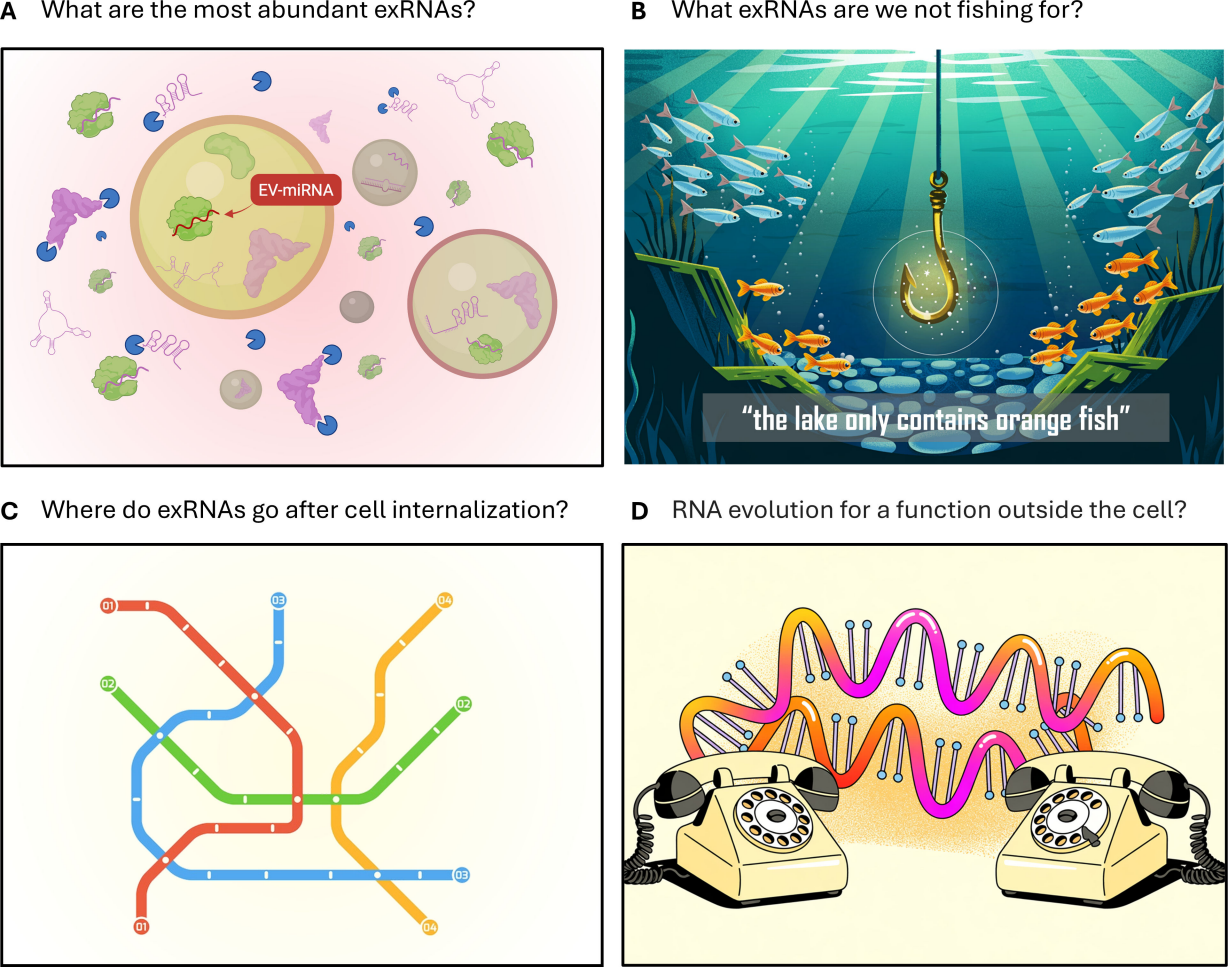
Four questions that could help expand the exRNA field in new directions. (**A**) What are the most abundant exRNA types both within and outside EVs? EV-miRNAs (red arrow) represent only a tiny fraction of the extracellular RNAome. On the one hand, miRNAs only represent a minor fraction of the vesicular RNAome. On the other hand, many exRNAs are nonvesicular, including ribonucleoprotein complexes and naked, unbound exRNAs. The abundance of these nonvesicular RNAs partially depends on their sensitivity or resistance to extracellular RNases (depicted in blue). (**B**) What exRNAs remain undetected (blue fish) by our current RNA sequencing protocols (yellow hook) due to technical biases? (**C**) What is the subcellular localization of the internalized exRNAs in the recipient cell? This can be important for certain proposed functions that require the exRNAs to reach the cytosol or the nucleus of the recipient cell, and not simply the surface or the endosomal network. (**D**) Could a primary function of certain RNA types (e.g., Y RNAs) be to act in the extracellular space? Some RNA types may be relics of the RNA world preserved due to their roles in intercellular communication pathways, whose functions cannot be understood if simply considering their existence inside of one cell. Figures were made using BioRender (**A**) or Ideogram 3.0 (**B, D**).

While we may not yet have a definitive answer to this question, we would like to propose four additional questions that might illuminate the path forward (Fig. 1).

### What RNA types are most abundant in the extracellular space both within and outside EVs?

To date, most analyses have focused on guide-acting small RNAs (miRNAs, siRNAs, short tRNA fragments) or messenger RNAs (mRNAs) where there is a mechanistic framework for how they could function and existing methodologies for validation, for example, with reporter assays. However, the data across diverse organisms show that other classes of RNA (tRNAs, Y RNAs, SRP RNA, vault RNAs, rRNAs) are often more abundant both inside and outside of EVs, and there remain few investigations into their transmission or function within recipient cells (Fig. 1A).

### What are we missing due to technical or conceptual limitations but that could be relevant to understanding exRNA functionality?

The most “unbiased” method to answer this question is sequencing, but the library generation protocols and methods of analysis will direct the answer. An example is the focus in the literature on fragments of tRNAs or Y RNAs in EVs based on sequencing, rather than a focus on full-length forms that are excluded when making the library and only become revealed by northern blots or specialized sequencing techniques (e.g., ARM-seq, TGIRT-seq, and hydro-tRNA-seq) (8, 15). RNA modifications and the balance between RNases and RNase inhibitors can also strongly influence what gets sequenced and what is not. For a recent review on biases in small RNA sequencing and their impact on exRNA profiling, see reference 36. Finally, the analysis methods define what is kept as useful information or discarded. For example, often, sequences that map to repetitive regions in the genome are thrown out, even though this is a ubiquitous and poorly understood source of exRNA across eukaryotes (11, 37, 38) that is also prevalent in host-pathogen exRNA interactions (17). We are still stuck in many ways looking for what we already understand and expect (Fig. 1B).

### What exRNAs are most likely to play a role in intercellular communication based on their proposed mechanism of action, stability, and abundance?

**Fig 2:**
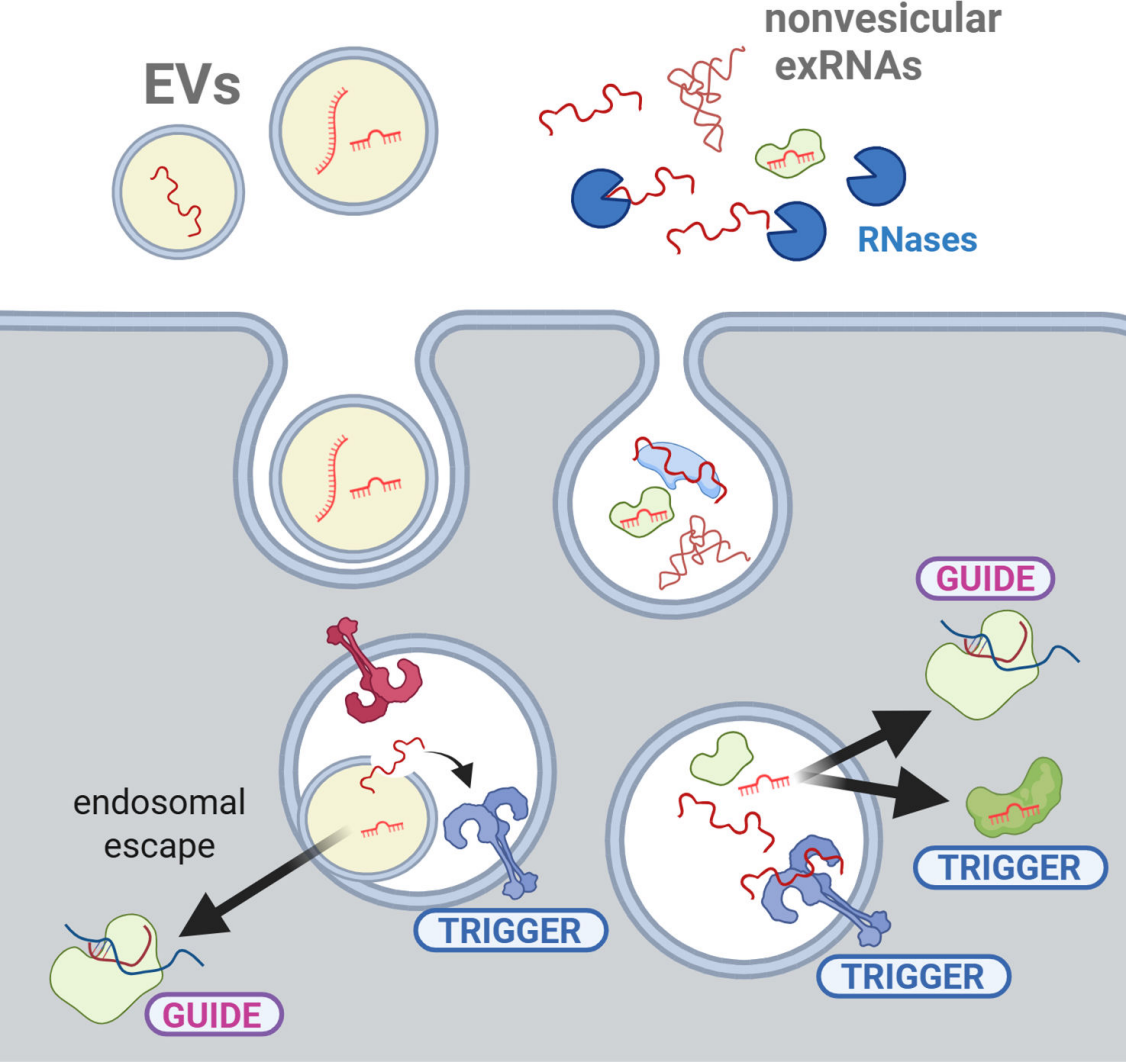
Guides or triggers? Subcellular localization restricts or enables functional possibilities. Both vesicular and nonvesicular exRNAs can be internalized by endocytosis if not degraded by extracellular RNases. Once inside endosomes, vesicular RNAs need to escape into the cytosol to recognize specific targets in a sequence-dependent manner (“guides”; e.g., miRNAs and siRNAs that function in gene silencing with Argonaute proteins). This is thought to occur by fusion of the EVs with the endosomal membrane. However, vesicular RNAs can still be functional in the absence of efficient endosomal escape, for example, by activating RNA sensors localized inside endosomes (“triggers”). The same considerations apply to nonvesicular exRNAs, which are directly exposed in the endosomal lumen. Note that exRNAs can also act as triggers in the cytosol if they are recognized by a protein or by another nucleic acid in a structure-dependent but sequence-independent manner (e.g., RIG-I). Thus, “guides vs triggers” is a distinction based on the mechanism of action rather than in subcellular localization, but sequence-dependent mRNA recognition is thought to occur exclusively in the cytosol.

While the focus has been on miRNAs due to the existing mechanistic framework for how they function in the cytoplasm of cells, the framework for how they make it to the cytoplasm after entering the cell is lacking. If EVs are internalized by endocytosis, EV-miRNAs could be released into the cytosol of a recipient cell after the fusion of the EV lipid bilayer with the endosomal membrane (39). However, this seems to be quite an inefficient process in mammalian studies (12, 40–42), and more work is needed to understand when/where/how escape can occur in different contexts and how small RNAs subsequently end up in the right complexes (43). A simpler mechanism for some exRNAs could occur within endosomes (Fig. 2), where exRNAs can activate RNA-specific Toll-like receptors (TLRs) (44). This has been shown for vesicular tRNA-derived fragments (45, 46) that are prevalent and abundant across EVs from Bacteria, Archaea, and Eukarya. It is even tempting to speculate that microbiome-derived exRNAs might have shaped the evolution of mammalian endosomal RNA sensors. For example, a highly stable bacterial rRNA-derived fragment can be spontaneously internalized by murine immune cells, even when present in culture media as a naked RNA, and is a potent trigger of endosomal TLR13 (35) (Fig. 1C).

### Could there be important non-cell autonomous functions of some non-coding RNAs that have defined their evolution?

Over the last 50+ years, the field of RNA biology has built quantitative and mechanistic data on how diverse RNAs function inside cells. The field of exRNA has evolved after (and largely separate from) this. Based on this chronology, any role of an exRNA might be expected to be known already; the only details we have to work out are how it gets from donor to recipient. But, what if the functions of some RNAs can only be well understood if we account for their roles outside the cell? What have we been missing? For example, Y RNAs are abundant in extracellular samples (7, 11, 47), including in human biofluids (48–50), but their intracellular roles are still not fully understood despite being discovered more than 40 years ago. In fact, these RNAs were originally discovered in the extracellular space as the RNA component of a ribonucleoprotein particle targeted by self-reactive antibodies (51) (Fig. 1D).

Why is it worth pushing this field forward? The RNA world drove the evolution of life, and it would be bizarre for its innovative power to be restricted to within the cell membrane. Indeed, most aspects of life require interaction outside the cell, yet our understanding of the different roles of exRNAs in living systems is still incredibly limited. The exRNA field remains ripe for discovery if we can evolve our technologies and minds to build a foundation of knowledge that includes things we may not already expect.
